# Eye Movements Predict Recollective Experience

**DOI:** 10.1371/journal.pone.0002884

**Published:** 2008-08-06

**Authors:** Tali Sharot, Matthew L. Davidson, Meredith M. Carson, Elizabeth A. Phelps

**Affiliations:** 1 Wellcome Department of Imaging Neuroscience, Institute of Neurology, University College London, London, United Kingdom; 2 Department of Psychology, Columbia University, New York, New York, United States of America; 3 Department of Psychology, Drexel University, Philadelphia, Pennsylvania, United States of America; 4 Department of Psychology, New York University, New York, New York, United States of America; Victoria University of Wellington, New Zealand

## Abstract

Previously encountered stimuli can bring to mind a vivid memory of the episodic context in which the stimulus was first experienced (“remembered” stimuli), or can simply seem familiar (“known” stimuli). Past studies suggest that more attentional resources are required to encode stimuli that are subsequently remembered than known. However, it is unclear if the attentional resources are distributed differently during encoding and recognition of remembered and known stimuli. Here, we record eye movements while participants encode photos, and later while indicating whether the photos are remembered, known or new. Eye fixations were more clustered during both encoding and recognition of remembered photos relative to known photos. Thus, recognition of photos that bring to mind a vivid memory for the episodic context in which they were experienced is associated with *less* distributed overt attention during encoding and recognition. The results suggest that remembering is related to encoding of a few distinct details of a photo rather than the photo as a whole. In turn, during recognition remembering may be trigged by enhanced memory for the salient details of the photos.

## Introduction

Previously encountered stimuli can bring to mind a vivid memory of the episodic context in which the stimulus was first experienced. Alternatively, a stimulus can simply seem familiar; known to have been experienced earlier but does not bring to mind related details to further specify the memory. In both cases the stimulus is recognized as encountered previously, however the subjective recollective experience is vastly different. Studies using the remember/know paradigm, a method frequently used to examine the recollective experience [1], have demonstrated that a number of variables can selectively affect subjective reports of “remembering” (recognition accompanied by recollection of associative information), and “knowing” (familiarity based recognition). One such variable is attention. It has been suggested that while knowing involves automatic processing which depends on fluency, remembering depends on distinctive processing that requires more attention [2]. Consistent with this notion, divided attention tasks at study lead to large reductions in remembering with little effect on knowing [3], [4]. During recognition, however, divided attention tasks do not have as a robust effect on remembering [5], suggesting that during test less additional attentional resources are needed for remembering than during encoding.

While it has been established that remembering is more attention demanding than knowing (for a review see 5), it is unclear how the attentional resources are distributed during encoding and recognition of remembered stimuli. In this study we examine whether remembering and knowing are related to different patterns of allocation of overt attention as indicated by eye-movements (e.g., 6). Two possible patterns of attention allocation may be related to remembering. According to one hypothesis, given that remembering involves recognition accompanied by contextual information it is possible that the added attentional resources are used to encode and retrieve additional associated details. If this is the case, attention will be more disperse when encoding and retrieving remembered stimuli than known stimuli.

Alternatively, focusing attention on a few distinct details while viewing an event can produce deep encoding of those specific details, which may later provide robust cues to rely upon during recognition. This in turn may strengthen the recollective experience. Support for this hypothesis comes from the literature on emotion and memory. Emotion has been shown to enhance subjective remember responses [7], [8], as well as narrow attention during encoding [9]. Studies report that when observing an emotional slide eye fixations are focused primarily on the central, arousing, details of the stimulus, and less on peripheral details [10], [11]. It is possible that the narrowing of attention during encoding is related to the boost in the recollective experience, and that this relation is not necessarily specific to emotional stimuli.

Past studies have suggested that eye movements during recognition reflect memory; during test sampling rates of previously encountered stimuli are decreased compared to novel stimuli, indicating memory of the “old” stimuli [12], [13]. Additionally, different eye movement patterns have been reported to distinguish repeated scenes from manipulated scenes [12], [14]. To date, it is unknown if eye movements are also a reliable marker of the subjective experience that accompanies recognition, and if a specific pattern of eye movements during encoding is subsequently related to the recollective experience. To examine this, eye movements were recorded while participants viewed emotional and neutral photos, and later while they indicated whether the photos were “remembered”, “known”, or new. We then related the number of eye fixations, and the inter fixation distance, which conveys the degree of clustering of fixations, to the participants' response.

## Methods

### Participants

Seventy eight participants (age 18–35) were recruited through posted advertisements. Seven were eliminated from the analysis because they did not have a sufficient number of trials (at least 5 per critical condition) to allow reliable analysis of the eye movement data. Two participants were eliminated because of difficulties in recording their eye movements. The remaining sixty-nine participants were included in the analysis. Eye movements were recorded during encoding for thirty-nine participants, and during recognition for all sixty-nine participants. All participants gave written informed consent and were paid for their participation. The study was approved by the University Committee on Activities Involving Human Subjects (UCAIHS) at New York University.

### Stimuli

Stimuli consisted of 70 negatively arousing photos, and 70 neutral photos, selected from the International Affective Photo Series (IAPS), based on their standard scores for emotional arousal and emotional valence [15], and from our own set of neutral pictures, to equate the two sets for the presence of humans and visual complexity [8]. All photos were rated in a previous study for valence and arousal [8]. Valence was rated on a scale from 1 (positive) to 9 (negative). Neutral photos were rated as neutral (M = 3.75, SD = 1.07) and emotional photos as negative (M = 7.69, SD = 0.52); t (10) = 14.23, P<0.0001. Arousal was rated on a scale from 1(not at all arousing) to 9 (very much arousing). Neutral photos had lower arousal ratings (M = 3.03, SD = 0.83), than emotional photos (M = 6.79, SD = 1.15); t (11) = 10.67, P<0.0001.

### Apparatus

ETS-PC System ASL 504 eye tracking device was used (Applied Science Laboratories).

### Behavioral Task

Participants went through an incidental encoding task consisting of 120 trials, which included presentation of 60 neutral photos and 60 emotional photos. Each photo was presented for 2 s (eye movements were recorded at this time), after which the participant had 2s to rate the photo for visual complexity, and then a fixation cross appeared for 6 s. The trials were separated into four blocks of 30 trials each.

Forty-five minutes after the encoding session the participants were given the “remember/know” recognition test [1], [16] in which subjects are asked to classify previously experienced stimuli as either (i) vividly “remembered” stimuli that evoke a specific memory for the episodic context in which the stimuli was experienced or as (ii) a stimuli that is simply “known” to have been experienced earlier or (iii) new. There were four practice trails.

The recognition test included the presentation of forty old negatively arousing photos, forty old neutral photos, ten new negatively arousing photos, and ten new neutral photos. As the aim of this study was to compare eye movements related to remember and know response a greater number of old photos were included, so to obtain a sufficient number of remember and know responses in each category that will allow reliable data analysis. The exact proportion was determined according to a previous study [8], in which the pattern of behavioral results was found to be similar to that of a study using an equal number of old and new photos [7]. Old and new photo sets were counterbalanced across participants. Stimuli were presented in a random order on a computer screen at a viewing distance of 50 cm. Each trial consisted of the presentation of a photo for 2 s (during which time eye movements were recorded), followed by 2 s to indicate a response (either new, remember, or know) by pressing the appropriate button on a button box, and finally a fixation cross appeared for 4 s. The trials were separated into four blocks of 25 trials each.

### Recording of Eye-Movements

Participants placed their head on a chin-rest with right eye positioned 0.5 m from screen and eye camera. The IR illuminations was positioned to illuminate the right eye. Images of the pupil and corneal reflection were captured at 60 HZ. Photos (size = 5.5″×5.5″) were displayed at the pre-set position marked by a 9-point matrix on the monitor. Before the beginning of the session eye calibration was performed using the 9-point matrix. Re-calibration was performed between blocks as needed. Eye movements were recorded during the 2 s photo display. At that time 120 gaze positions were captured at equal intervals (approximately every 17 ms).

### Data Analysis

Eyenal analysis software was used to transform raw gaze positions to fixation points according to the following algorithm: the beginning of a fixation point was where six sequential gaze positions had a standard deviation not exceeding 0.5 visual degrees, the end of a fixation point was where the next three sequential gaze positions were at least one visual degree apart from initial fixation position, fixation point was defined at the average point of beginning and end. Statistical analysis was conducted using both the average number of fixations per condition, and the inter fixation distance, which is the average distance between two sequential fixation points. The inter fixation distance conveys the degree of clustering of fixations. A decrease in inter-fixation distance will indicate enhanced clustering of fixations.

## Results

Remember responses (R) were measured as the proportion of old items receiving a remember response minus the proportion of new items receiving this response. R responses were greater for emotional stimuli than neutral stimuli; P<0.001 (**Table 1**). Because know responses are mathematically constrained by remember responses independent know scores (K) were calculated, indexing the probability that an item received a know response given that it did not receive a remember response: K = Khit/(1-Rhit)−Kfa/(1-Rfa) [17]. K scores did not differ for emotional and neutral stimuli. False alarms for know responses were greater for emotional than neutral photos; P<0.05 (**Table 1**). Overall accuracy (hit rates minus false alarm rates collapsed across R and K responses) did not differ for neutral and emotional pictures. The behavioral results replicated previous results of studies using similar paradigms [7], [8].

**Table 1:** Proportion of remember and know responses for old and new emotional and neutral items.

Remember responses				Know responses			
Emotional		Neutral		Emotional		Neutral	
Old	New	Old	New	Old	New	Old	New
.57	.00	.40	.00	.32	.03	.49	.01

### Eye Movement Data

A 2 (type of photo: emotional/neutral) by 2 (response: “remember”/“known”) repeated measures ANOVA was conducted on (1) inter fixation distance, and (2) number of eye fixations per photo. Analysis was conducted on correct “remember” and “know” responses only, as accuracy was very high (0.9), and our main interest was in comparing “remember” and “know” responses. All means and standard deviations are presented in **Table 2**.

**Table 2:** Average inter-fixation distance (measured in eye tracking units, which are equal to 1/20 inch), and average number of fixations for remember and know responses for emotional and neutral items.

	Inter fixation distance				Number of fixations			
	Encoding		Test		Encoding		Test	
	Remember	Know	Remember	Know	Remember	Know	Remember	Know
Emotional	32.4 (.8)	33.4 (1.1)	29.2 (.6)	30.0 (.6)	5.5 (.1)	5.4 (.2)	4.9 (.1)	5.0 (.1)
Neutral	34.1 (.8)	35.1 (1.2)	29.3 (.7)	31.0 (.6)	5.4 (.1)	5.3 (.1)	4.8 (.1)	4.9 (.1)

(sem).

#### Encoding

Inter-fixation distance was characterized by a main effect of response F (1, 38) = 3.96, P<0.05, with smaller inter-fixation distances (i.e., more clustered fixations) for subsequently “remembered” photos than “known” photos. This result suggests, that the narrowing of eye fixations during encoding is predictive of subsequent “remembering”. Additionally, there was a reliable main effect of emotion F (1, 38) = 6.18, P<0.025, with inter-fixation distance smaller for emotional photos than neutral photos. The results of a within subjects t-test indicated that inter-fixation distance for emotional photos which were later “remembered” was smaller than for neutral photos later “remembered” t (38) = 2.74, P<0.005. No other comparisons, or interaction, were found to be significant.

Number of eye fixations was characterized by a main effect of emotion F (1, 38) = 5.32, P<0.05, with average number of fixations greater for emotional photos than neutral photos. No other comparisons, or interaction, were found to be significant.

These results suggest that enhanced clustering of fixations at encoding is related to subsequent “remembering”, and that emotional scenes elicit enhanced sampling rates and clustering relative to neutral scenes.

#### Test

Inter-fixation distance was characterized by a main effect of response F (1, 68) = 11.36, P<0.001, with inter-fixation distance smaller (i.e., more clustered fixations) for “remembered” photos than “known” photos. The results of a within subjects t-test indicate that inter-fixation distance for “remembered” neutral photos was smaller than for “known” neutral photos t (68) = 2.96, P<0.005. No other comparisons, or interaction, were found to be significant.

Number of eye-fixations were characterized by a main effect of response F (1, 68) = 4.3, P<0.05, with average number of fixations greater for “known” photos than “remembered” photos. There was no significant main effect of emotion or interaction.

**Figure 1:**
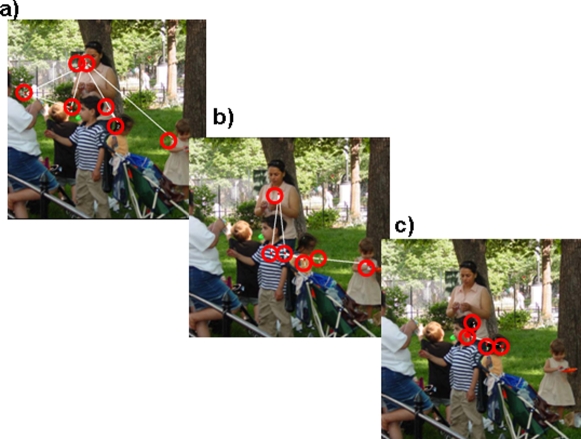
Example of eye movement patterns. An example of eye movements from three participants. (a) A participant viewing a new scene. (b) A participant viewing a scene for the second time and classifying it as “known”. (c) A participant viewing the scene for a second time and classifying it as “remembered”. The examples demonstrate fewer and more clustered fixations (smaller inter-fixation distance) for “remembered” photos than “known” photos and new photos.

These results suggest that reduced sampling rates of previously encountered scenes, and enhanced clustering of fixations, is indicative of “remembering” relative to “knowing” (**Fig. 1**), and that the effect is similar for emotional and neutral stimuli.

## Discussion

It has been previously shown that remembering requires more attentional resources than knowing, possibly because remembering is related to deeper processing of the stimulus [5]. However, it was unknown if attention is allocated differently when processing remembered and known stimuli. The current study reveals that a rich recollective experience is related to a *narrowing* of overt attention during both encoding and recognition.

During encoding, photos subsequently remembered elicited more clustered eye-fixations than photos subsequently known. This was indicated by smaller distances between fixations. These findings suggest that the additional resources thought to be needed during encoding of remembered stimuli [5], are channeled towards deep encoding of a specific part of the scene. Focusing overt attention on particular, and presumably significant, details may produce strong cues that can prompt a remember response during recognition.

During recognition, eye fixations were more clustered when viewing remembered photos than known photos. In addition, remembered photos elicited fewer fixations than photos simply known to have been encountered. A decrease in the sampling rate of previously encountered scenes, compared to novel scenes, has been reported in the past [12]. Here, we provide evidence suggesting that reduced sampling rates does not only signify repeated exposure, but is also a reliable measure of the subjective sense of recollection that accompanies recognition. It is possible that in the absence of recollection participants use the allotted time for additional sampling of the photo in search of recollective cues before settling on a know judgment. Greater clustering of fixations during recognition of remembered photos relative to known photos suggest that recognition accompanied by a sense of remembering may be driven by strong memory for part of the scene, rather than the scene as a whole.

Past studies have emphasized the implicit nature of eye movement patterns during recognition. Eye movements have been found to reflect previous exposure even in the absence of explicit awareness of the change [12], and regardless of whether the task required intentional retrieval [18]. In fact, differential viewing of studied stimuli can be observed well in advance of explicit identification of that stimulus [18]. The present study is consistent with these past results in suggesting that eye movements provide a particularly sensitive measure of memory, and expand them by identifying changes in eye movement patterns that are related to the subjective feeling accompanied by recognition, as measured by explicit behavioral responses.

The current findings can be interpreted within dual process and single process theories of recognition memory. From the perspective of dual process models, which suggest that two distinct processes (recognition and familiarity) underlie recognition [5], the current results indicate that the recollection component of recognition is related to narrowed overt attention during encoding and recognition. By single process models, in which recognition is assumed to be based on a global measure of memory strength, the results suggest that narrowed attention during encoding and recognition is associated with high recognition confidence. According to either model the findings indicate that a strong recollective experience (related both to high confidence and remember responses) is related to narrowed attention during encoding and test.

Although our data suggests that eye movements are a reliable indicator of the recollective experience during both encoding and recognition, the results do not speak of causation. It is possible that clustered fixations result in enhanced remembering, or that a third factor such as distinctiveness leads to both narrowing of overt attention and an enhancement in the recollective experience. We speculate that overt attention is captured by salient details of a scene, as a result those details are deeply encoded and then retrieved during recognition to produce a strong sense of remembering.

One category of salient details that can capture attention are details conveying emotional information. Our findings indicate that emotion both modulated eye movements during encoding, and enhanced the subjective recollective experience. First, emotional scenes elicited more eye fixations during study than neutral scenes. An emotional scene often holds critical information and thus may result in additional sampling. Second, when viewing an emotional scene eye fixations were more clustered relative to neutral scenes. This finding is in accord with Easterbrook's hypothesis (1959), according to which emotion decreases the span of cues an organism is attending to [9]. The results are consistent with previous findings showing that emotion elicits more eye-fixations to the central aspects of a scene, but not to peripheral aspects [11], [19]. Thus, emotion may be related to greater sampling of a scene during encoding, but the samples are taken from a narrow part of the scene, presumably one that bears emotional information. There was no interaction between emotion and memory on any measures of eye movements. Taken together, the findings suggest that the narrowing of overt attention by emotion is one factor that may lead to a heightened feeling of remembering. However, the relation between narrowing of attention and enhanced feeling of remembering is not unique to emotional stimuli.

To our knowledge, this is not only the first attempt to understand the mechanisms underlying the recollective experience by examining eye-movements, but also the first study to relate eye movements during encoding to subsequent memory. The results of this study suggest that recognition of a scene that brings to mind a vivid memory for the episodic context in which it was experienced is associated with *less* distributed overt attention during encoding and recognition. This may indicate that the additional attentional resources required for remembering are used for deep encoding of a local part of the scene, and during recognition the subjective sense of recollection is trigged by enhanced memory for that part of the scene.

## References

[1] Tulving E (1985). Memory and consciousness.. Canadian Psychology.

[2] Gardiner JM, Gregg VH, Karayianni I (2006). Recognition memory and awareness: occurrence of perceptual effects in remembering or in knowing depends on conscious resources at encoding, but not at retrieval.. Mem Cognit.

[3] Curran T (2004). Effects of attention and confidence on the hypothesized ERP correlates of recollection and familiarity.. Neuropsychologia.

[4] Mangels JA, Picton TW, Craik FI (2001). Attention and successful episodic encoding: an event-related potential study.. Brain Res Cogn Brain Res.

[5] Yonelinas AP (2002). The nature of recollection and familiarity: a review of 30 years of research.. Journal of Memory and Language.

[6] Posner ML (1980). Orienting of attention.. Q J Exp Psychol.

[7] Ochsner KN (2000). Are affective events richly recollected or simply familiar? The experience and process of recognizing feelings past.. Journal of Experimental Psychology General.

[8] Sharot T, Delgado MR, Phelps EA (2004). How emotion enhances the feeling of remembering.. Nature Neuroscience.

[9] Easterbrook JA (1959). The effect of emotion on cue utilization and the organization of behavior.. Psychology Review.

[10] Christianson SA, Loftus EF, Hoffman H, Loftus GR (1991). Eye fixations and memory for emotional events.. Journal of Experimental Psychology: Learning Memory and Cognition.

[11] Wessel I, van der Kooy P, Merckelbach H (2000). Differential recall of central and peripheral details of emotional slides is not a stable phenomenon.. Memory.

[12] Ryan JD, Althoff RR, Whitlow S, Cohen NJ (2000). Amnesia is a deficit in relational memory.. Psychological Science.

[13] Althoff RR, Cohen NJ (1999). Eye-movement-based memory effect: A reprocessing effect in face perception.. Journal of Experimental Psychology: Learning, Memory, and Cognition.

[14] Ryan JD, Cohen NJ (2004). The nature of change detection and on-line representations of scenes.. Journal of Experimental Psychology: Human Perception and Performance.

[15] Lang PJ, Bradley MM, Cuthbert BN (1999). International affective picture system (IAPS): Instruction manual and affective ratings.

[16] Rajaram S (1993). Remembering and knowing: two means of access to the personal past.. Memory and Cognition.

[17] Yonelinas AP, Jacoby LL (1994). Dissociations of processes in recognition memory: effects of interference and of response speed.. Canadian Journal of Experimental Psychology.

[18] Hannula DE, Ryan JD, Tranel D, Cohen NJ (2007). Rapid onset relational memory effects are evident in eye movement behavior, but not in hippocampal amnesia.. Journal of Cognitive Neuroscience.

[19] Christianson SA, Loftus EF (1991). Remembering emotional events: The fate of detailed information.. Cognitive & Emotion.

